# Analysis of Prescribing Practices in the Dermatology Outpatient Department of a Tertiary Care Teaching Hospital

**DOI:** 10.7759/cureus.37910

**Published:** 2023-04-20

**Authors:** Bapugouda Patil, Jyoti Patil, Leela Hugar, Gurudatta Moharir

**Affiliations:** 1 Pharmacology and Therapeutics, Bijapur Lingayat District Educational Association (BLDEA) (Deemed to be University) Shri BM Patil Medical College Hospital and Research Center, Vijayapura, IND; 2 Pharmacology and Therapeutics, Dr.Ulhas Patil Medical College and Hospital, Jalgaon, IND

**Keywords:** opd- outpatient department, corticosteroids, antifungals, antihistaminics, polypharmacy, potential drug-drug interactions, prescription writing

## Abstract

Introduction

The practice of appropriately prescribing and delivering pharmaceuticals to the right patient for the diagnosis, prevention, and treatment of diseases is referred to as "rational drug usage". Patients should receive pharmaceuticals that are appropriate for their clinical needs, given in doses that meet their needs, for long enough periods of time, and for the least amount of money possible. Minimizing drug therapy costs without sacrificing therapeutic effectiveness, avoiding unnecessary adverse medication reactions and drug-drug interactions, and improving therapeutic care while encouraging patient adherence are the main objectives of rational drug usage. The present study was planned to assess the current prescribing practices in the dermatology outpatient department of a tertiary care hospital.

Materials and methods

A prospective descriptive study was conducted in the department of dermatology at a tertiary care teaching hospital after receiving permission from the institutional ethics committee. The study was conducted from November 2022 to February 2023 and followed the WHO recommendation for sample size. A total of 617 prescriptions were analyzed thoroughly.

Results

Regarding the demographic profile of the 617 prescriptions, 299 were male and 318 were female. The patients had diverse diseases, with the most common being tinea infection (57 cases, 9%) and acne vulgaris (53 cases, 8.5%), followed by scabies (38 cases, 6%), urticaria, and eczema (30 cases, 5%). Twenty-six (4%) prescriptions were not written in capital letters, 86 (13%) prescriptions did not mention the route of drug administration, and the consultant's or physician's name and signature were missing in 13 (2%), and six (1%) prescriptions, respectively. None of the prescriptions were written using the generic names of the drugs. Polypharmacy was observed in 51 (8%) prescriptions. Moreover, potential drug-drug interactions were identified in 12 (1.9%) instances. The most prescribed drugs were antihistaminics, with 393 (23%) prescriptions. Antifungal drugs were the second most prescribed, with 291 (17%) prescriptions. Corticosteroids were also commonly prescribed, with 271 (16%) prescriptions. Antibiotics were prescribed in 168 (10%) cases; other drugs were prescribed in 597 (35%) cases, including retinoids, anti-scabies drugs, antileprotic drugs, moisturizers, sunscreens, etc.

Conclusion

The study highlighted the prescription errors in writing the drugs in capital letters, mentioning the dose, route, and frequency of drugs, etc. It provided insight into the common diseases in dermatology and routine prescribing patterns and addressed the frequency of polypharmacy and drug-drug interactions.

## Introduction

Appropriate prescribing practice and delivery of the pharmaceuticals to the right patient as a part of preventive care, diagnosing a medical condition, and treating the disease are referred to as "rational drug use" [1]. Patients should receive appropriate pharmaceuticals for their clinical requirements in proper strength and dose for a clinically sufficient period of time with the least possible cost [2]. Minimizing drug therapy costs without compromising therapeutic effectiveness, avoiding adverse medication reactions and drug-drug interactions, and improving therapeutic care while encouraging patient adherence to the treatment are the main objectives of rational drug usage [3]. The prescription of drugs is one of the common tools used by physicians to treat illness, lessen signs and symptoms, and stop the development of new diseases.

Prescription of drugs requires expertise in diagnostics, an understanding of common medications and their therapeutic effects, adverse effects, and drug interactions, a grasp of the fundamentals of clinical pharmacology, communication abilities, and the capacity to weigh the pros and cons of treatment [4]. Adverse drug events can occur due to medication errors, with a reported 11% of such events being attributed to medication errors according to a study conducted by Gandhi et al [5]. Among patient safety issues, medication errors are among the most frequent, and among medication errors, prescribing errors are particularly common [6]. A prescribing error can produce an unintentional reduction in the likelihood of timely and effective treatment and/or an increase in harm compared to generally accepted medical practices [7].

Periodic evaluation of drug prescribing and administration procedures is crucial in order to quantify errors and identify potential solutions, as emphasized in the reference [8]. Neville et al. propose a classification system for prescription errors based on the degree of nuisance they pose to dispensing operations, with type A errors posing a potentially serious risk to the patients. Type B errors are those that cause significant inconvenience to the patient, requiring pharmacist consultation with the prescriber before dispensing. Type C errors cause minor inconveniences that can be resolved by consulting other pharmacists at the dispensary level. Type D errors include those that are trivial in nature, such as spelling mistakes or missing patient details, or those that do not impede the execution of dispensing the drugs [9].

Diseases affecting the skin are on the rise, and they are one of the major health issues worldwide. Skin disorders stood at the eighteenth rank for a global disease burden and the fourth rank for nonfatal disease burden in 2010 [10]. Skin disorders constitute 2% of outpatient department consultations globally [11]. Some of the common dermatological disorders in India are acne, pyoderma, urticaria, scabies, dermatitis, and cutaneous fungal infections [12]. Unfortunately, there are several issues with the prescription patterns of drugs for these conditions in India: the use of antibiotics for fungal infections, therapeutic duplication of drugs, unnecessary use of multivitamins, and irrational and illogical drug combinations [13,14]. To minimize the potential risks associated with multiple medications, the World Health Organization (WHO) has recommended that physicians limit the average number of drugs per prescription to 2.0 [15]. Polypharmacy is defined as the concurrent use of five or more medications by a patient, as the chances of adverse drug reactions and drug interactions can increase as the number of drugs per prescription increases. Polypharmacy also leads to decreased patient compliance and increases the cost of the treatment [15]. A study conducted by Thawani et al. found that there is a significant prevalence of polypharmacy in dermatology prescriptions [16].

## Materials and methods

Aims and Objectives

to analyze the prescription pattern in patients visiting the dermatology outpatient department, identify the errors in the prescriptions, and identify the potential drug-drug interactions.

A prospective descriptive study was conducted in the department of dermatology at Shri BM Patil Medical College Hospital and Research Center, a tertiary care teaching hospital in Vijaypura, India, in collaboration with the department of pharmacology after receiving permission from the institutional ethics committee of the Bijapur Lingayat District Educational Association (BLDEA) (deemed to be a university). The approval letter number is BLDE(DU)/IEC/807-A/2022-23. The study was conducted from November 2022 to February 2023 and followed the WHO recommendation for sample size. WHO suggests a minimum sample size of 600 in any cross-sectional studies to analyze and describe current prescribing practices [17]. A total of 617 prescriptions were collected from patients who visited the outpatient department of dermatology during the study period.

The collected prescriptions were analyzed according to the guidelines of the WHO core drug prescribing indicators and criteria for investigating drug use in health facilities [17]. The following criteria were analyzed: Demographic data like patients’ name, age, sex, and UHID number; physician data like the physician's name, registration number, and signature; prescription data like diagnosis, legibility, capital letters, generic name, and spelling; medication data like the number of drugs per prescription, dose, route, and frequency; antibiotic data like the number of antibiotics per prescription

All 617 prescriptions were thoroughly scrutinized for the above-mentioned prescribing indicators, and errors in the prescribing pattern were identified and noted. A drug-drug interaction (DDI) was checked using the drugs.com interaction checker. The prescriptions containing fixed drug combinations were checked for drug interactions individually.

## Results

The collected data were analyzed using Microsoft Excel (version 2302), and descriptive statistics were used to analyze the results. Additionally, percentages and averages are compared with other findings. In the demographic distribution of the 617 patients, 299 were male and 318 were female. Their age and gender distribution are presented in Table 1.

**Table 1:** Age and sex distribution of the patients

Age	Male (n=293)		Female (n=318)	
	Number of patients	Percentage	Number of patients	Percentage
0-10 years	39	13%	35	11%
11-20 years	47	16%	61	19%
21-30 years	53	18%	59	18%
31-40 years	38	13%	62	19%
41-50 years	42	14%	46	14%
51-60 years	27	10%	35	11%
Above 60 years	47	16%	20	8%

The patients had diverse diseases, the most common being tinea infection (57 cases, 9%) and acne vulgaris (53 cases, 8.5%), followed by scabies (38 cases, 6%), urticaria, and eczema (30 cases, 5%). Other diseases, such as melasma, psoriasis, vitiligo, atopic dermatitis, and leprosy, among others, were also reported, as described in Table 2.

**Table 2:** Disease distribution of the patients

Diagnosis	Number	Percentage (%)	Diagnosis	Number	Percentage (%)
Vitiligo	20	3	Keloid	8	1
Hyperkeratotic dermatosis	12	2	Hansen’s disease	17	3
Scabies	38	6	Polymorphous light eruption	13	2
Tinea cruris with tinea corporis	57	9	Pompholyx	8	1
Contact dermatitis	20	3	Prurigo simplex	9	1
Psoriasis	25	4	Tinea incognito	6	1
Pruritus corporis	12	2	Varicella	9	1
Urticaria	30	5	Pityriasis rosea	10	2
Melasma	26	4	Folliculitis	9	1
Acne vulgaris	53	9	Candidal intertrigo	6	1
Alopecia areata	16	3	Atopic dermatitis	15	2
Herpes zoster	6	1	Pityriasis alba	19	3
Eczema	29	5	Others	138	22
Telogen effluvium	6	1	Total	617	

None of the prescriptions lacked information on the patient’s name, age, sex, and unique health identification (UHID) number, as the outpatient department (OPD) slips were generated electronically. Moreover, all prescriptions were legible, and diagnosis and frequency of drug administration were mentioned. None of the prescriptions had spelling errors. However, 26 (4%) prescriptions were not written in capital letters, 86 (13%) prescriptions did not mention the route of drug administration, and the consultant's or physician's name and signature were missing in 13 (2%), and six (1%) prescriptions, respectively. None of the prescriptions were written using the generic name of the drugs, and 33 (5%) prescriptions did not contain the medical council registration number of the treating physician. The details of prescription errors are outlined in Table 3.

**Table 3:** The details of prescription errors

Parameters	Errors (%)	Parameters	Errors (%)
Patient name	0	Capitals	26 (4%)
Date	0	Dose/ Strength	14 (2%)
Age	0	Frequency	0
Sex	0	Route of drug administration	86 (13%)
Consultant name	13 (2%)	Correct spelling	0
UHID No	0	Generic name	671 (100%)
Diagnosis	0	Consultant sign	6 (1%)
Legible	0	Consultant registration number	33 (5%)

An analysis of medication data revealed that a total of 1720 medications were prescribed for the 617 patients, averaging 2.7 drugs per prescription. Of the total prescriptions, 168 had at least one antibiotic. Polypharmacy was observed in 51 (8%) prescriptions. Moreover, potential drug-drug interactions were identified in 12 (1.9%) instances. The details of potential drug-drug interactions are presented in Table 4.

**Table 4:** The details of potential drug-drug interactions (DDI)

Drug combination	Severity of DDI	Number	Details of DDI
Benzoyl peroxide and adapalene	Moderate	3	A combination of retinoids and keratolytic agents may cause excessive irritation/drying of the skin.
Doxepin and hydroxyzine	Moderate	2	Agents with anticholinergic properties may have additive effects when used in combination. Excessive parasympatholytic effects may result in paralytic ileus, hyperthermia, heat stroke, and the anticholinergic intoxication syndrome
Azithromycin and fluconazole	Moderate	5	Can increase the risk of irregular heart rhythms.
Rivaroxaban and pentoxifylline	Moderate	2	The use of pentoxifylline has been associated with bleeding and/or prolongation in prothrombin time. The risk of bleeding may be increased by concomitant treatment with anticoagulants

**Figure 1:**
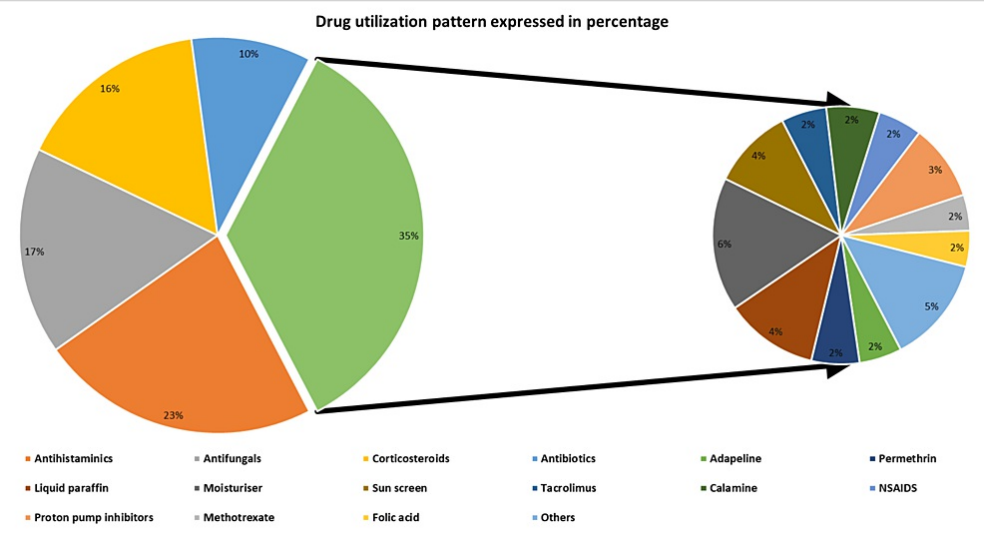
Drug utilization pattern

**Figure 2:**
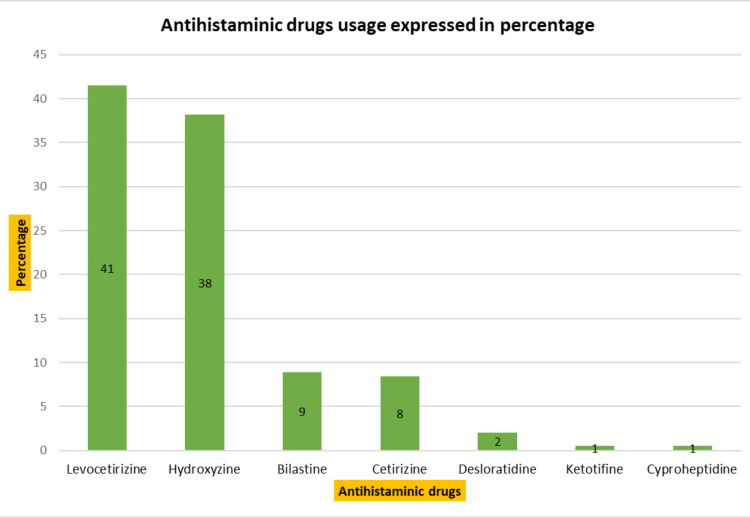
Antihistaminic drugs usage (n=393)

**Figure 3:**
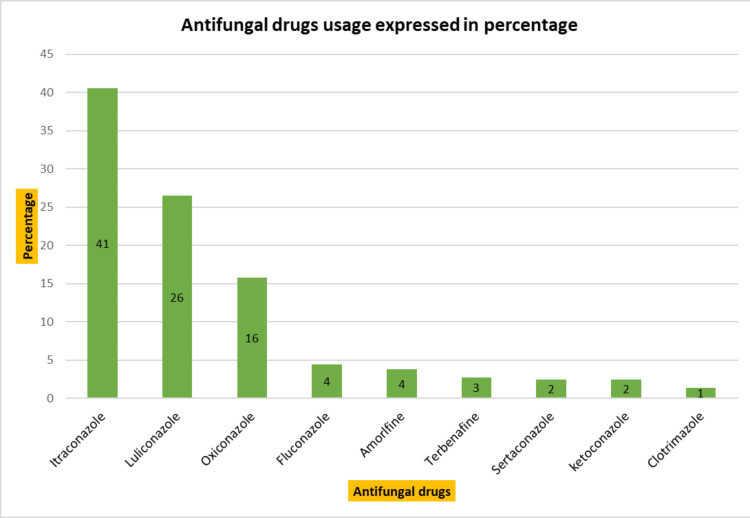
Antifungal drugs usage (n= 291)

**Figure 4:**
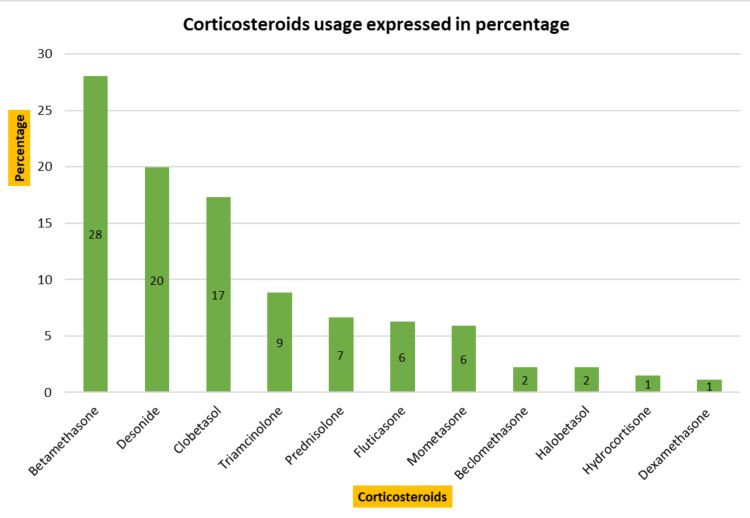
Corticosteroids usage (n=271)

The outpatient medication utilization analysis is presented below. The most commonly prescribed drugs were antihistaminics, with 393 (23%) prescriptions. The most frequently prescribed antihistaminics were levocetirizine and hydroxyzine, followed by cetirizine, bilastine, and others. Antifungal drugs were the second most prescribed, with 291 (17%) prescriptions. Itraconazole and luliconazole were the preferred antifungal drugs, followed by oxiconazole and fluconazole. Corticosteroids follow the list after antifungals, representing 271 (16%) of prescriptions. Preference is in the following order: betamethasone, desonide, clobetasol, triamcinolone, and others. Antibiotics were prescribed in 168 (10%) cases, commonly including benzoyl peroxide, azithromycin, and clindamycin. Other drugs were prescribed in 597 (35% of the cases), including retinoids, anti-scabies drugs, antileprotic drugs, moisturizers, sunscreens, etc. A detailed description of drug utilization is depicted in Figure 1, and the utilization of antihistaminics, antifungals, and corticosteroids is presented in Figures 2-4, respectively.

## Discussion

A prescription is a written order from a qualified and registered medical practitioner that specifies the details of the required medication or treatment for the patient’s well-being. It reflects the doctor's expertise and approach to patient care, considering their physical condition and financial situation, as well as availability, affordability, quality, rationality, and completeness. A drug-utilization study analyzing prescriptions encompasses all these aspects to provide a comprehensive analysis [18].

Prescription errors and prescribing faults are significant concerns in medication errors that can affect patient safety and healthcare quality. Prescription errors refer to mistakes made during prescription writing while prescribing faults encompass various issues, such as irrational prescription, using inappropriate medications, under- or overprescribing, and ineffective prescriptions that arise from errors in medical judgment or decision-making concerning treatment and monitoring. To achieve appropriate prescribing, minimizing errors during the prescription writing process and actively working towards better prescribing practices are essential for optimal outcomes [19,20].

The study analyzed 617 prescriptions, all with complete patient information, clear writing, diagnosis, and drug frequency mentioned, without spelling errors. However, 4% were not in capital letters, 13% did not mention the drug administration route, and 2% and 1% lacked the physician’s name and signature, respectively. None used generic drug names, and 5% did not include the treating physician's medical council registration number. The disease distribution was diversified, with tinea infection (9%) and acne vulgaris (9%) being the most common diseases, followed by scabies and eczema in patients visiting dermatology OPD. The results were similar to the study conducted by Anuj Kumar Pathak et al. [18]: acne vulgaris was the most common, followed by fungal infections and eczema. The average number of drugs used per prescription was 2.7, similar to the study conducted by Indurkar et al., which found 2.72 drugs per prescription [20]. Polypharmacy was observed in 51 (8%) of the prescriptions. Antihistaminics accounted for 23% of all prescriptions, followed by antifungal drugs at 17%, corticosteroids at 16%, and antibiotics at 10%. Levocetirizine and hydroxyzine were the most commonly prescribed antihistaminics, while itraconazole and luliconazole were the most frequently prescribed antifungal drugs. Betamethasone and desonide were the most commonly prescribed corticosteroids, and benzoyl peroxide, azithromycin, and clindamycin were the most commonly prescribed antibiotics. Similar results were shown by Indurkar et al. [20], who presented antihistaminics, followed by antifungals and corticosteroids, as the most commonly prescribed drugs.

## Conclusions

The studies analyzing prescriptions carry very good information regarding disease distribution, rational drug use, and the pattern of handwritten prescriptions. The present study highlighted the prescription errors in writing the drugs in capital letters, mentioning the dose, route, and frequency of drugs, and mentioning the consultant’s name and signature. It provided insight into the common diseases in dermatology and routine prescribing patterns and addressed the frequency of polypharmacy and drug-drug interactions. The study helps dermatologists reduce common and simple prescribing errors and, with polypharmacy, potential drug-drug interactions, and commonly used medications; hence, it improves the overall therapeutic benefit to the patient.
